# Alcohol-clozapine synergism triggering rhabdomyolysis at subtoxic levels: a case report and literature review

**DOI:** 10.3389/ftox.2025.1692362

**Published:** 2025-12-17

**Authors:** Wenzhen Zhou, Yuxin Sun, Mingcheng Dai, Senlin Ma, Qiuxin Lu, Junwei Qian, Zhaoming Shang, Kangshuai Zhou, Mingquan Chen, Xiaofei Jiang

**Affiliations:** 1 Department of Emergency, Huashan Hospital, Shanghai Institute of Infectious Disease and Biosecurity, Fudan University, Shanghai, China; 2 Department of Emergency, Huashan Hospital, Fudan University, Shanghai, China; 3 Department of Infectious Diseases, Shanghai Key Laboratory of Infectious Diseases and Biosafety Emergency Response, National Medical Center for Infectious Diseases, Huashan Hospital, Fudan University, Shanghai, China; 4 Department of Cardiology, Huashan Hospital, Fudan University, Shanghai, China

**Keywords:** clozapine, rhabdomyolysis, drug synergism, ethanol, adverse drug reaction

## Abstract

**Background:**

While excessive oral intake of clozapine is known to cause severe complications, this case report highlights that even conventional doses of clozapine ingested after alcohol consumption–without reaching toxic concentrations–can lead to the rare complication of rhabdomyolysis when synergistic effects occur between the two substances.

**Case presentation:**

We report a case of a 39-year-old male patient who presented with impaired consciousness 1 day after consuming 500 g liquor followed by ingestion of 8 clozapine tablets (200 mg total). Toxicology screening upon admission indicated subtoxic levels of clozapine. Laboratory findings confirmed rhabdomyolysis, acute kidney injury, and myocardial damage. During hospitalization, the patient underwent hemopurification and fluid resuscitation. His consciousness improved significantly, accompanied by marked improvement in creatine kinase (CK) levels, renal function, and cardiac enzymes. At 15-day follow-up, renal function, CK levels, and cardiac enzymes had returned to within normal limits.

**Conclusion:**

In patients consuming clozapine after alcohol intake, dynamic monitoring of CK and myoglobin should be implemented regardless of whether toxicology results are within normal limits. Early implementation of blood purification can effectively disrupt the rhabdomyolysis-renal injury cascade, thereby securing a critical therapeutic window for clinical intervention.

## 1 Introduction

Clozapine, a second-generation antipsychotic drug classified as a dibenzodiazepine derivative, directly inhibits the brainstem reticular formation and exhibits potent sedative-hypnotic effects. First synthesized in Bern, Switzerland in 1958, it entered clinical use in Europe during the 1970s. China commenced domestic production in 1976, and by the early 1980s, clozapine was designated as a first-line treatment for schizophrenia (Ruan et al., 2024). The therapeutic dosage ranges from 25 mg to 50 mg daily, with gradual titration up to 300–400 mg/day if tolerated (Ashton et al., 2000). Excessive use correlates with multiple adverse effects, including agranulocytosis (cumulative incidence 2.5%) (Nofziger et al., 2025), cardiomyopathy (estimated 0.1%–1.0%) (Curto et al., 2016), and rare instances of acute interstitial nephritis (0.04%) (Hunter et al., 2009), seizures (0.183%) (Bleich et al., 2024), and rhabdomyolysis (0.19%) (Kotake et al., 2023). Conventionally, toxicity is associated with serum concentrations >1,000 ng/mL (Shigeev et al., 2013), however, severe complications occur even with standard oral dosing (Jansman et al., 2015; Patteet et al., 2020). Alcohol—a commonly co-ingested substance—shares cytochrome P450 metabolic pathways with clozapine. The precise mechanism underlying their synergistic induction of rhabdomyolysis remains unelucidated (De Bartolomeis et al., 2022). Crucially, whether muscle cell necrosis persists after complete ethanol metabolism lacks substantive investigation. Determining if alcohol-clozapine interactions provoke rhabdomyolysis at subtoxic or therapeutic doses represents an urgent clinical priority. This report details the clinical course of a patient developing rhabdomyolysis following co-ingestion of therapeutic-dose clozapine and ethanol. It demonstrates that ethanol-triggered pathological cascades can breach traditional concentration thresholds even after ethanol clearance, providing critical evidence for risk assessment in schizophrenia management.

## 2 Case presentation

A 39-year-old male freelancer with a history of depression (and no other regular prescription medications reported) and irregular clozapine use presented to our emergency department with 24 h of unconsciousness. According to family members, 24 h prior to admission, he concurrently ingested 500 g liquor and eight clozapine tablets (200 mg). He subsequently developed marked agitation with disorientation, initially dismissed as alcohol intoxication. After 24 h, he progressed to unresponsiveness, necessitating emergency transport via EMS.

Physical examination revealed a temperature of 36.3 °C, a heart rate of 107 beats per minute, a respiratory rate of 17 breaths/min, and blood pressure of 145/105 mmHg. The patient was comatose with a Glasgow Coma Scale score of 6 (E1V2M3), exhibiting facial flushing, bilateral 2-mm pinpoint pupils with sluggish light reflexes, and a pronounced ethanol odor emanating from clothing. Neurological assessment revealed bilateral extensor plantar responses.

Laboratory tests are shown in Supplementary Table 1. The ECG showed sinus tachycardia; however, the head CT scan and abdominal ultrasound were unremarkable.

**FIGURE 1:**
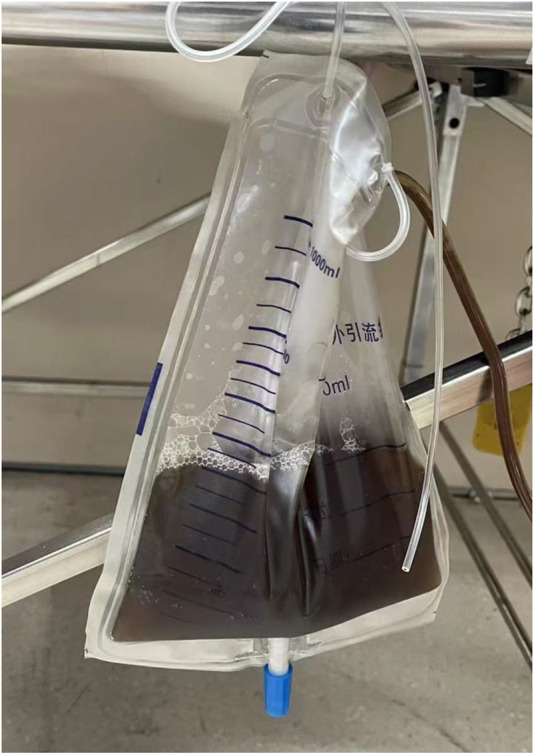
Tea-colored urine sample obtained via urinary catheterization on admission.

Toxicology analysis on admission confirmed a subtoxic but clinically significant clozapine level (650 ng/mL) with no other drugs or alcohol detected. Urinary catheterization produced 300 mL of tea-colored urine (Figure 1), supporting diagnoses of clozapine-induced rhabdomyolysis, renal and hepatic impairment, and myocardial injury. Combined blood purification (CVVHDF + hemoperfusion) and fluid resuscitation were started on day 1. Persistent oliguria with worsening rhabdomyolysis and renal function required continued extracorporeal detoxification through day 2. By day 6, clozapine levels fell to 80 ng/mL with improved consciousness, oliguria persisted, prompting ongoing CVVHDF. A shift to polyuria occurred around 2 weeks, with concomitant improvement in renal and muscle parameters. At discharge, serum creatinine showed a drop to 296 μmol/L from 711 μmol/L, while creatine kinase and myoglobin normalized. The patient was discharged stabilized. At 15-day follow-up, renal function, urine output, and mental status had fully recovered (Detailed lab data in Supplementary Table 1).

## 3 Discussion

The diagnosis of rhabdomyolysis in this patient was primarily attributed to the synergistic effect of alcohol and clozapine. However, other common causes of rhabdomyolysis were considered and ruled out. There was no recent history of trauma, crush injury, or intense physical exertion reported by the family or evident on physical examination. Electrolyte imbalances, which can precipitate muscle damage, were not present upon admission. Common infections were unlikely given the patient’s normal temperature and unremarkable initial infectious workup. Genetic myopathies were deemed improbable in the absence of a suggestive personal or family history. Furthermore, toxicology screening was negative for other classic myotoxic substances such as drugs and toxins. The temporal relationship between the co-ingestion of alcohol and clozapine and the onset of symptoms, coupled with the exclusion of these alternative causes, strongly supports the conclusion of a drug-induced, synergistic rhabdomyolysis.

This case demonstrates that clozapine, even at a therapeutic dose yielding serum concentrations above the therapeutic range yet below the conventional toxic threshold of 1,000 ng/mL (Shigeev et al., 2013), can trigger severe rhabdomyolysis. This provides compelling evidence that life-threatening complications may occur at concentrations traditionally considered safe or subtoxic (particularly with concurrent alcohol ingestion) challenging the clinical reliance on serum levels alone to assess poisoning severity, a caution underscored by Patteet et al. who documented severe clinical outcomes in 3 patients with the clozapine level (350–600 ng/ml) well below the conventional toxic threshold (Patteet et al., 2020).

The accumulation of alcohol and its metabolites significantly impacts human health. Numerous studies indicate that alcohol metabolism is influenced by the activities of ADH and ALDH enzymes (Edenberg et al., 2019; Edenberg and McClintick, 2018). Particularly, variants in ALDH2 lead to acetaldehyde accumulation, increasing blood acetaldehyde levels up to sixfold and causing target organ damage (Rwere et al., 2024; Thoudam et al., 2024). Although the patient’s blood ethanol was undetectable on arrival—consistent with the 24-hour delay since ingestion—the lingering effects of acetaldehyde accumulation cannot be overlooked, especially when clozapine is taken after alcohol consumption, which may enhance its toxicity (Monroy-Jaramillo et al., 2022; Ortega-Vázquez et al., 2021). This interaction may stem from shared genetic pathways involving dopamine regulation and Dopamine Receptor D4 (DRD4) in treatment-resistant schizophrenia (TRS), where alcohol potentiates clozapine’s adverse effects (Rajagopal et al., 2018). A study of 48 TRS patients (Ortega-Vázquez et al., 2021) found that drinkers carrying the CYP1A2*1C/*1C genotype had significantly higher risk of adverse reactions (OR = 7.9, p = 0.016), particularly CNS effects such as somnolence, consistent with this case. Thus, alcohol intake must be considered in patients taking clozapine (Johnson and Seneviratne, 2014; Mattila, 1990; Weathermon and Crabb, 1999).

Furthermore, it is crucial to recognize the inherent and broad-spectrum toxicity of alcohol itself. Beyond its pharmacokinetic interactions with clozapine (Monroy-Jaramillo et al., 2022; Ortega-Vázquez et al., 2021), alcohol is a well-established independent risk factor for multi-organ damage, including rhabdomyolysis, acute kidney injury, and cardiomyopathy, as highlighted in a comprehensive review on alcohol abuse disorder fatality (Argo et al., 2024). Consequently, the dynamic monitoring of CK and myoglobin we advocate for serves a dual purpose: it detects damage arising from the alcohol-clozapine synergism, but it is also critically relevant for assessing the direct myotoxic and nephrotoxic effects of alcohol itself, which may have been the initiating event.

Why did this patient exhibit such high serum clozapine levels despite a therapeutic dose? Ethanol likely played an inducing role. Both ethanol and clozapine are metabolized by CYP450 enzymes. Although the patient’s blood ethanol was undetectable on arrival, earlier ingestion could have allowed it to bind with high affinity to the active site of CYP3A4 (Fraser, 1997; Jin et al., 2011), competitively inhibiting clozapine metabolism. As ethanol is a known CYP3A4 inducer, this interaction significantly delays clozapine clearance (Monroy-Jaramillo et al., 2022), leading to accumulation even at moderate doses—consistent with this case. Mouse studies confirm that co-administration of ethanol and clozapine for 24 h results in unusually high serum clozapine (Romanova et al., 2018). Thus, even low-dose clozapine requires vigilance for drug-interactions, particularly with alcohol.

Adverse effects of clozapine overdose commonly include agranulocytosis, acute interstitial nephritis, myocarditis, and seizures (Bishara and Taylor, 2014; De las Cuevas et al., 2024; Elias et al., 1999). Rhabdomyolysis is rare, occurring in approximately 0.19% of cases (Kotake et al., 2023), yet may lead to severe complications such as acute kidney injury (AKI) (Béchard et al., 2022). The pathophysiology of AKI in rhabdomyolysis includes intratubular obstruction by myoglobin casts, direct tubular cytotoxicity, and renal vasoconstriction. Filtered myoglobin precipitates in the acidic and concentrated urine of the distal tubule, forming obstructive casts with Tamm-Horsfall protein, which is a central event in the development of acute tubular necrosis (Bosch et al., 2009).

Current evidence is largely based on case reports and retrospective analyses. A 2011 review of 18 acute clozapine poisonings identified only one case of rhabdomyolysis (Gawlikowski et al., 2011). Another study described a 36-year-old with schizophrenia and alcohol use disorder who developed altered mental status 4 h after ingesting 125 mg clozapine. On admission, CK was 9,899 U/L, clozapine level 3,177 μg/L, and ethanol undetectable. CK normalized after drug cessation and fluid resuscitation (Jansman et al., 2015). In our case, clozapine toxicity was promptly recognized through history-taking, yet AKI still developed. After 17 days of blood purification and fluid therapy, the patient’s parameters largely returned to normal. Early aggressive intervention may improve outcomes (Keltz et al., 2014).

The severity of clozapine intoxication correlates with elevated CK. Using the Naranjo probability scale (Naranjo et al., 1981), a score of 6 indicated a probable association between rhabdomyolysis and clozapine overdose in this patient. The mechanism remains unclear (De Bartolomeis et al., 2022), but may involve: 1) modulation of JAK-STAT inflammatory signaling, potentially contributing to muscle injury (Zhang et al., 2025); 2) increased muscular membrane permeability via 5-HT2A receptor activation (Meltzer et al., 1989); and 3) nigrostriatal pathway blockade leading to hyperkinesis and rigidity (Devarajan and Dursun, 2000). Despite multiple hypotheses, definitive molecular targets and pathways are lacking, underscoring the need for basic molecular and pharmacogenomic investigations.

The complexity of this case lies in the sequential ingestion of alcohol and clozapine, highlighting a critical alert for clinicians: even with negative toxicology or safe drug levels, a 24-hour alcohol history must be reviewed and dynamic CK/myoglobin monitoring initiated in clozapine-treated patients. During the follow-up after discharge, the patient recalled that due to his recent start-up failure, he had developed suicidal ideation. The patient expressed profound trust in the medical intervention, deep apprehension regarding alcohol-clozapine co-ingestion, and consented to the use of their case for educational purposes to prevent harm to others. This report urges reevaluation of the clinical implications of subtoxic concentrations—when synergistic factors are present, conventional safety thresholds may conceal life-threatening risks.

A limitation of this report is the incomplete long-term psychiatric history, including the definitive indication for clozapine, which could not be fully elucidated during emergency management. Additionally, baseline somatometric data (height/weight) were not available from the emergency records.

## 4 Conclusion

In patients consuming clozapine after alcohol intake, dynamic monitoring of CK and myoglobin should be implemented regardless of whether toxicology results are within normal limits. Early implementation of blood purification can effectively disrupt the rhabdomyolysis-renal injury cascade, thereby securing a critical therapeutic window for clinical intervention.

## Data Availability

The original contributions presented in the study are included in the article/Supplementary Material, further inquiries can be directed to the corresponding authors.
